# An Optimized Feeder‐Free Method for Isolation and Expansion of Functional ILC2s Isolated From Human Peripheral Blood

**DOI:** 10.1155/jimr/7688318

**Published:** 2026-08-27

**Authors:** Lina K. Andersson, Miriam Bollmann, Agnieszka Lastowska, Isabel Haupt, Lisi Wagener, Anders Nguyen, Johan Svensson, Linda C. Johansson, Mattias N. D. Svensson

**Affiliations:** ^1^ Department of Rheumatology and Inflammation Research, Sahlgrenska Academy, Institute of Medicine, University of Gothenburg, Gothenburg 41346, Sweden, gu.se; ^2^ Department of Paediatrics, Skåne University Hospital, Lund University, Malmö, Sweden, lu.se; ^3^ Department of Medical Biochemistry and Cell Biology, Sahlgrenska Academy, Institute of Biomedicine, University of Gothenburg, Gothenburg 41390, Sweden, gu.se; ^4^ SciLifeLab, Institute of Biomedicine, University of Gothenburg, Gothenburg, Sweden, gu.se

## Abstract

Innate lymphoid cells (ILCs) are tissue‐resident innate counterparts of T cells. Group 2 ILCs (ILC2s) produce cytokines such as IL‐5 and IL‐13 in response to alarmins, including IL‐25 and IL‐33 and play a key role in type 2 immunity. Lately, ILC2s have become a major focus across several research areas, including asthma, allergic lung inflammation, atopic dermatitis, and liver fibrosis. However, ILC2s are rare in human peripheral blood, making their isolation and expansion for functional studies challenging. Existing methods commonly rely on flow‐cytometry‐based cell sorting, which requires large sample volumes, expensive reagents, and considerable hands‐on time. Moreover, in vitro expansion protocols often depend on feeder cell lines and activating alarmins. Here, we present a simple and efficient approach for isolating and expanding human ILC2s from peripheral blood, without the need for flow‐cytometry‐based cell sorting, feeder cells, or external alarmins. This method yields pure and viable ILC2s from both large and small blood volumes, enabling the use of limited or rare patient samples. Expanded ILC2s responded robustly to alarmin stimulation by proliferating and producing signature cytokines and retained functionality after cryopreservation and reculture. Together, this method provides a user‐friendly and cost‐effective alternative to current human ILC2 isolation and expansion protocols.

## 1. Introduction

Innate lymphoid cells (ILCs) were identified just over a decade ago as the innate counterparts of T cells. Together with natural killer (NK) cells, they constitute the family of ILCs, a group of lymphocytes characterized by the absence of antigen‐specific receptors and rapid cytokine responses. Based on their cytokine profiles, transcription factor expression, and effector functions, ILCs are divided into five main subsets: NK cells, ILC1s, ILC2s, ILC3s, and lymphoid tissue inducer (LTi) cells. As part of the innate immune system, ILCs act as early responders during immune activation, reacting to inflammatory cues and epithelial‐derived alarmins such as IL‐15, IL‐1β, IL‐23, IL‐25, and IL‐33, which are released upon infection or tissue damage. Upon activation, ILCs secrete effector cytokines that recruit and activate other immune cells to promote pathogen clearance [1–4].

ILC2s, the functional equivalents of Th2 cells, play a central role in type 2 immunity by contributing to helminth defense and orchestrating inflammation through the secretion of the type 2 cytokines IL‐4, IL‐5, IL‐9, and IL‐13 [1, 3–5]. Consequently, ILC2s have been implicated as key mediators in several type 2 cytokine‐driven diseases, including asthma, allergic lung inflammation, atopic dermatitis, and liver fibrosis, as well as other inflammatory and autoimmune conditions [5–10]. ILC2s are predominantly tissue‐resident cells found at barrier surfaces such as the lung, skin, and intestine and are therefore rare in human peripheral blood, where they account for only 0.05%–0.1% of total leukocytes [4, 11]. Moreover, human ILC2s express markers that overlap with Th2 cells and other ILC subsets, requiring careful panel design for accurate phenotyping. They are typically defined as cells lacking expression of common lineage markers (e.g., CD3, CD19, and CD14) and expressing the surface markers CD127, CD161, and CRTH2 (chemoattractant receptor‐homologous molecule expressed on Th2 cells), as well as high expression of the transcription factor GATA3 [4, 12]. Among these, CRTH2 is one of the few surface receptors that reliably distinguishes ILC2s from other ILC subsets and is therefore widely used for their identification and isolation [4, 12–14].

Despite the existence of these defining markers, isolation and expansion of human ILC2s remain technically challenging. Most current studies isolate ILC2s from human peripheral blood using magnet‐based negative or positive selection combined with flow‐cytometry‐based cell sorting [13, 15, 16]. Due to the low number of cells that are usually obtained during isolation, ILC2s are typically expanded in vitro with feeder cell lines, such as mouse OP9 cell lines, in the presence of recombinant cytokines and alarmins, including IL‐25 and IL‐33, to promote survival and proliferation [13, 17]. However, these protocols have several limitations. Because ILC2s are rare in human blood, flow‐cytometry‐based sorting generally requires large sample volumes and complex antibody panels, making the process time‐consuming, cost‐intensive, and unsuitable for rare patient materials. In addition, the need to culture and maintain feeder cell lines adds consumable costs, complexity, and potential variability. Furthermore, while alarmins can substantially enhance ILC2 proliferation, they also have the potential to alter the cellular phenotype, promoting a more activated and inflammatory profile that may confound downstream functional assays [18, 19]. Access to high‐speed sorters and specialized reagents further constrains the use of these methods in routine experimental settings.

In this study, we describe a refined and accessible but robust method for the isolation and in vitro expansion of human ILC2s from peripheral blood that overcomes several of these challenges. Our approach yields pure, viable, and functionally competent ILC2s from both large‐scale (buffy coat) and small‐volume (9 mL) blood samples, without the need for flow‐cytometry‐based sorting, feeder cells, or exogenous alarmins such as IL‐25 or IL‐33 during expansion. The small‐scale format is particularly advantageous for clinical studies, enabling the use of limited or precious patient samples without compromising cell purity or viability. This optimized method provides a scalable, cost‐effective, and accessible alternative to current ILC2 isolation methods, facilitating downstream mechanistic and translational investigations.

## 2. Material and Methods

### 2.1. Materials

For detailed information regarding materials, including catalog numbers, vendors, and antibody clones, used in the experimental procedures described below, please see Table S1.

### 2.2. Human ILC2 Isolation

Buffy coats used for large‐scale ILC2 isolation were obtained from healthy human blood donors at the blood bank at Sahlgrenska University Hospital in Gothenburg, Sweden. All buffy coats were obtained anonymously, and therefore, no ethical approval was required according to the Swedish legislation section code 4§ 3p SFS 2003:460 (Law on Ethical Testing of Research Relating to People).

Blood for small‐volume isolation was obtained from four healthy female and four healthy male human donors under ethical permits 2019‐00205 and 2022‐02545‐02. Blood was collected into lithium heparin tubes, and peripheral blood mononuclear cells (PBMCs) were isolated using SepMate PBMC Isolation Tubes containing Ficoll‐Paque density gradient media according to the manufacturer’s instructions.

Isolated PBMCs from either whole blood or buffy coats were counted using a LUNA‐FX7 cell counter. For large‐scale isolation, cells were diluted to 2 × 10^8^ cells/mL in 1 mL of Dulbecco′s phosphate‐buffered saline (DPBS) supplemented with 1 mM EDTA (Invitrogen) and 2% heat‐inactivated fetal bovine serum (FBS). For small‐scale isolation, 1 × 10^7^ cells were used in a total volume of 250 µL. ILC2s were then isolated using the EasySep Human ILC2 Isolation Kit with the EasySep Magnet according to the manufacturer’s instructions. Due to the lower input of cells for small‐scale isolation, manufacturer’s instructions were adjusted, and ¼ of recommended volume was used for kit components, including FC‐blocker, CRTH2‐PE Positive Selection Cocktail, Release PE Positive Selection Cocktail, Releasable RapidSpheres, Release Buffer, ILC2 Isolation Cocktail, and Dextran RapidSpheres. Incubation times and volumes in other steps were kept according to the original instructions. For details on component volumes for large‐scale and small‐scale isolation, see Table S2.

### 2.3. Human ILC2 In Vitro Expansion

Isolated ILC2s were seeded into a 96‐well (Nunclon Delta Surface round‐bottom 96‐well plate) in 200 µL of ILC2 culture media containing MEMα GlutaMax, 100 units/mL penicillin, 100 ng/mL streptomycin, 10% human serum (Type AB), 50 µM 2‐mercaptoethanol, 50 µg/mL gentamicin, supplemented with 20 ng/mL recombinant human IL‐2, IL‐7, and IL‐15. ILC2 culture media were refreshed daily by carefully removing ~100 µL of media and replacing with 100 µL of new preheated ILC2 culture media, supplemented with 40 ng/mL of IL‐2, IL‐7, and IL‐15. ILC2s were split before reaching a cell count of 300,000 cells per well (~1.5 × 10^6^/mL). ILC2s were counted every 3 to 4 days beginning 1 week after initial isolation to ensure that the cells were not disturbed during the early expansion phase. Cell viability was determined using acridine orange/propidium iodide (AO/PI) staining on a LUNA‐FX7 automated cell counter, with PI‐positive cells considered nonviable.

### 2.4. ILC2 Cryopreservation

After 21 days of culture, ILC2s, which were not used for functional testing, were cryopreserved. This was achieved by resuspending cells in 100% heat‐inactivated FBS in a 1.8 mL cryotube and then dropwise adding an equal volume of freezing media consisting of 20% dimethyl sulfoxide (DMSO) in FBS. The cryovial was placed in a Corning CoolCell freezing container and stored at −80°C. After 24 h, the cells were transferred to −150°C for long‐term storage. For experiments in this study, ILC2s were cryopreserved for up to 6 months, after which they were thawed by carefully adding preheated ILC2 culture media dropwise to the cryovial. Subsequently, the cell solution was carefully added to 10 mL of preheated ILC2 culture media and then rested at room temperature for 10 min before washing in DPBS. After washing, cells were resuspended in ILC2 culture media without cytokines and rested for 2 h before addition of cytokines: 20 ng/mL recombinant human IL‐2, IL‐7, and IL‐15. ILC2s were then cultured, as described above.

### 2.5. Flow Cytometry for ILC2 Purity and Sorting

The purity of isolated and expanded ILC2s was determined by flow cytometric analysis. Cells were stained extracellularly with the following anti‐human antibodies: BV605‐conjugated CD45, AF700‐conjugated CD161, and a PerCp‐Cy5.5‐conjugated lineage marker cocktail including CD19, CD3, CD14, CD11c, CD303, TCRγδ, TCRαβ, CD34, and FcεRIα. Viability was determined by staining with Fixable Viability Dye eFluor 506. After surface staining, cells were fixed and permeabilized with the eBioscience Foxp3/Transcription Factor Staining Buffer Set for intracellular staining with AF647‐conjugated GATA3 and PE‐CF594‐conjugated T‐bet. Stained cells were analyzed on a Beckman Coulter CytoFLEX. For information regarding clones and catalog numbers, see Table S1. Flow cytometry data were analyzed using FlowJo V.10 software. Fluorescence minus one (FMO) controls are used to define negative and positive stainings can be found in Figure S1 along with a full gating strategy in Figure S2.

For flow‐cytometry‐based cell sorting, cells were stained with the following anti‐human antibodies BV605‐conjugated CD45, AF700‐conjugated CD161, and a PerCp‐Cy5.5‐conjugated lineage marker cocktail including CD19, CD3, CD14, CD11c, CD303, TCRγδ, TCRαβ, CD34, and FcεRIα. Viability was determined by staining with Fixable Viability Dye eFluor 506. Cells were sorted into 100% FBS using a BD FACS Aria Fusion Cell sorter. The sorted cells were then recultured as described above.

### 2.6. Expression of Activation Markers in Freshly Isolated and Cultured ILC2s

ILC2s isolated from the buffy coat were divided into four equal fractions. One part (day 0) was stained with the following anti‐human antibodies: BV605‐conjugated CD45, AF700‐conjugated CD161, APC‐H7‐conjugated CD25, BB515‐conjugated ICOS, BV421‐conjugated KLRG1, PE‐conjugated ST2 (IL‐33R), and a PerCpCy5.5‐conjugated lineage marker cocktail including CD19, CD3, CD14, CD11c, CD303, TCRγδ, TCRαβ, CD34, and FcεRIα. Viability was determined by staining with Fixable Viability Dye eFluor 506.

The remaining three fractions were seeded into individual wells of a sterile 96‐well round‐bottom plate (Nunclon Delta Surface) in 200 µL of ILC2 culture media (incl. IL‐2,‐7, and ‐15 at 20 ng/mL) supplemented with either no additional alarmins (unstimulated), recombinant human IL‐25 (20 ng/mL), or recombinant human IL‐33 (20 ng/mL). The culture medium was refreshed daily, as described before. Following culture, ILC2s were counted as described above and stained with the same panel as that used for freshly isolated cells.

Stained cells were analyzed on a Beckman Coulter CytoFLEX, and flow cytometric data were analyzed using FlowJo. FMO controls used to define positive and negative staining populations are shown in Figure S3 along with a full gating strategy in Figure S4. Geometric mean fluorescence intensity (gMFI) values used for normalization to freshly isolated cells (day 0) are provided in Table S5.

### 2.7. Proliferation Assay

ILC2s were stained with CellTrace Violet according to the manufacturer’s instructions. The cells were then resuspended in ILC2 culture media and seeded into a sterile 96‐well round‐bottom plate (Nunclon Delta Surface) with 20,000 cells per well. Cells were incubated at 37°C for at least 10 min before adding stimulants into each well with a final concentration of 20 ng/mL of either IL‐12, IL‐23, IL‐25, or IL‐33. Cells were thereafter incubated for 4 days, after which cells were stained with Fixable Viability Dye eFluor 506 and analyzed on a Beckman Coulter CytoFLEX. Data were analyzed using FlowJo V10. The full gating strategy is found in Figure S5.

### 2.8. Quantification of Cytokines and Expression of Activation Marker

To assess cytokine production from cultured cells, 5000 ILC2s were seeded in a Nunclon Delta Surface round‐bottom 96‐well plate in either ILC2 culture media or ILC2 culture media supplemented with either IL‐12, IL‐23, IL‐25, or IL‐33. Cells were incubated for 3 days, after which the supernatant was collected for analysis using the LEGENDplex HU Th Cytokine Panel (12‐plex). Following the manufacturer’s instructions, supernatants from each stimulation condition were used to quantify the following cytokines: IL‐5, IL‐13, IL‐9, IL‐10, IFN‐γ, IL‐17A, IL‐17F, IL‐4, and IL‐22, using the Beckman Coulter CytoFLEX. LEGENDplex software was used for analysis.

Because expanding ILC2s secreted IL‐5 at levels exceeding the detection range of the LEGENDplex assay, IL‐5 concentrations in culture supernatants were also quantified using the ELISA MAX Standard Set Human IL‐5, following the manufacturer’s instructions.

Last, stimulated (IL‐25 or IL‐33) ILC2s were stained with an antibody panel including BV605‐conjugated CD45, BB515‐conjugated ICOS, and PE‐conjugated ST2 (IL‐33R) in combination with Fixable Viability Dye eFluor 506. Stained cells were analyzed on a Beckman Coulter CytoFLEX, and FlowJo was used to analyze the obtained flow cytometric data.

### 2.9. RNA Extraction, cDNA Synthesis, and qPCR

RNA was isolated from ILC2s using the Norgen Total RNA Purification Plus Micro Kit according to the manufacturer’s instructions. RNA concentration was measured using a NanoDrop One, and cDNA synthesis was performed using the Bio‐Rad iScript cDNA synthesis kit. The expression of *GATA3*, *IFNG*, *IL13*, *IL17A*, *IL5*, *RORC*, *IL17RB*, *IL1RL1*, and *TBX21* was analyzed using the Bio‐Rad SsoAdvanced Universal SYBR Green Supermix on a QuantStudio 6 Pro Real‐Time PCR System. Primer amplification efficiencies were determined by standard curve analysis of pooled cDNA from ILC2s. The amplification efficiency (*E*) was calculated from the slope of the standard curve: *E* = 10^−1/slope^. Gene expression was calculated using efficiency‐corrected quantification, thus accounting for gene‐specific amplification efficiencies with R (Version V.4.6.0) and normalization to GAPDH. The primers used are listed in Table S3.

### 2.10. Statistics and Reproducibility

Statistical analyses were performed using GraphPad Prism (V10.6.1) software. An RM one‐way ANOVA with subsequent Dunnett’s post hoc for multiple comparisons was performed in cases if not stated otherwise. Data are presented as mean ± SEM unless stated otherwise. A comparison was considered significant if *p*  < 0.05. Cytokine concentrations measured with the LEGENDplex assay were calculated and extrapolated using a standard curve that was generated using known cytokine concentrations (*X*) and the corresponding median fluorescence intensity (MFI; *Y*). Data were fitted by nonlinear regression using a five‐parameter logistic (5PL) model with an asymmetry term. Regression was performed with 1/*Y* ^2^ weighting to account for heteroscedastic variance across the dynamic range.

## 3. Results

### 3.1. Optimization of a Sorter and Feeder‐Free Method for Isolation and In Vitro Expansion of Human ILC2

**Figure 1:**
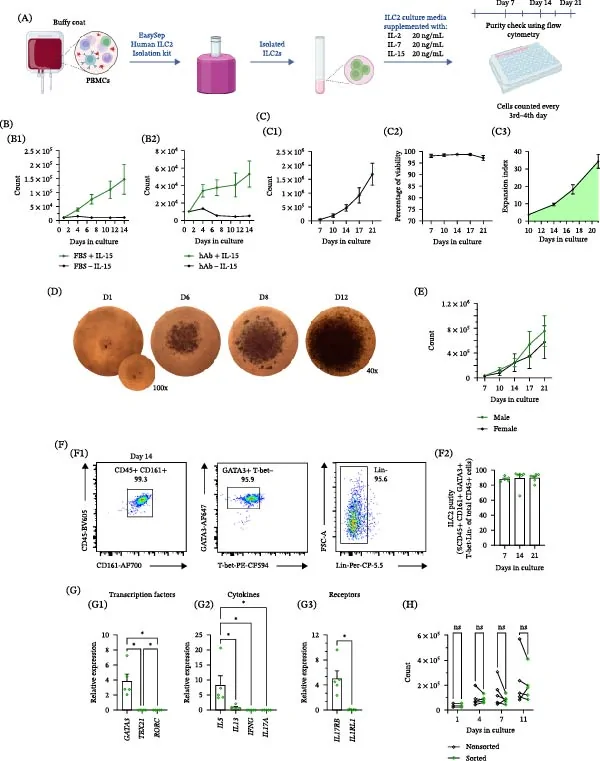
Optimization of a sorter‐ and feeder‐free method for isolation and in vitro expansion of human ILC2s. (A) Schematic representation of the workflow for human ILC2 isolation, in vitro expansion, and analysis from buffy coats (created with http://BioRender.com). (B) ILC2 growth from day 1 to day 14 in culture supplemented with fetal bovine serum (FBS; B1) or human AB serum (hAb; B2) in the presence of cytokines IL‐2 and IL‐7, with (+) or without (−) IL‐15 (*n* = 4 donors). (C) ILC2 growth, viability, and expansion index from day 7 to 21 for each time point counted (*n* = 6 donors). Expansion index was calculated from day 10 to 21. (D) Representative microscopic images of ILC2 cultures on days 1, 6, 8, and 12 after isolation (40x and 100x magnification). (E) ILC2 growth from day 7 to 21 comparing ILC2s derived from male (*n* = 4) and female (*n* = 4) donors. (F) Representative gating strategy used for assessment of ILC2 purity at different time‐points during culture. Representative gating from day 14 (F1). Lineage (Lin) markers include CD11c, CD14, CD19, CD303, CD34, CD3, FcεRI, TCRαβ, and TCRγδ. ILC2 purity was defined as the frequency of CD45+CD161+GATA3+T‐bet‐Lin‐ cells (*n* = 6 donors) (F2). (G) Relative expression of ILC‐associated transcription factors (G1), cytokines (G2), and receptors (G3) in unstimulated ILC2s (*n* = 5 donors). Gene expression was normalized to the housekeeping gene *GAPDH*. Statistical significance was determined using repeated‐measures one‐way ANOVA with Tukey’s post hoc for transcription factors and cytokines, and a paired *t*‐test for receptors. (H) Comparison of expansion of flow‐sorted and nonsorted ILC2s in culture (*n* = 5 donors). Significance was tested using a two‐way repeated‐measures ANOVA with subsequent Dunnett’s post hoc test. Data are presented as mean ± SEM.  ^∗^ *p* < 0.05, ns = nonsignificant.

The most common isolation and expansion protocols for human ILC2s rely on flow‐cytometry‐based cell sorting, followed by in vitro expansion utilizing mouse feeder cell lines [13, 15, 17]. To circumvent the need for cell sorter access as well as the utilization and maintenance of feeder lines such as OP9, we aimed to establish a user‐friendly and efficient method for isolating and expanding human ILC2s. To isolate ILC2s from human peripheral blood, we utilized a commercially available EasySep Human ILC2 Isolation Kit, which is based on an initial positive selection of CRTH2+ expressing cells, followed by negative selection of ILC2s. With a starting material of 2 × 10^8^ PBMCs, obtained from one buffy coat, we were able to obtain around 2000–3000 ILC2s, depending on the donor. This amount is expected based on the frequency of ILC2s in human peripheral blood and the utilized isolation kit [1, 13, 15]. After isolation, cells were immediately resuspended into preheated ILC2 culture media and seeded into a single well of a round‐bottom 96‐well plate (Figure 1A). Another feature of many published protocols for ILC2 expansion is the use of activating cytokines, for example, IL‐33 or IL‐25. Although these factors provide an additional expansion benefit, they are known to promote activation‐associated phenotypic changes in ILC2s, which may have implications for downstream applications [18, 19]. To limit activation‐associated phenotypic changes during expansion, we assessed different culture conditions that did not include IL‐33 or IL‐25. We found that a combination of cytokines IL‐2, IL‐7, and IL‐15 along with either 10% human serum‐type AB (hAB serum) or 10% FBS provided good growth conditions both in terms of viability and cell expansion (Figure 1B). Although FBS and hAB serum both yielded robust expansions, we decided to utilize hAB serum to minimize the risk of potential unspecific immune activation by factors present in bovine serum and to retain translational relevance. After optimization of expansion conditions, we optimized long‐term culture conditions. Cells were cultured for 7 days, with daily medium changes, after which cell number and viability were assessed every 3–4 days. Between days 7 and 21, hILC2s had expanded ~30 times and reached a yield between 5 × 10^5^ and 2 × 10^6^, which accounts for a ~600 times expansion in comparison to the day of isolation. Furthermore, mean viability was consistently high throughout the culture period, ranging between 97% and 99% (Figure 1C,D). No differences in expansion capacity were observed between male and female donors (Figure 1E).

Next, the purity of expanded ILC2s was assessed by flow cytometry at three time points (7, 14, and 21 days). ILC2s were characterized as cells expressing CD45, CD161, and GATA3 and being negative for a panel of lineage markers and the transcription factor T‐bet (Figure 1F). FMO controls for each marker were used to accurately distinguish positive and negative populations (Figure S1). The mean frequency of ILC2s remained around 90% across all assessed time points (Figure 1F), indicating the expansion of highly pure and viable ILC2s.

To further evaluate the purity of the isolated ILC2s, we analyzed mRNA expression of the transcription factors *TBX21*, *GATA3*, and *RORC*, the cytokines *IFNG*, *IL5*, *IL13*, and *IL17A*, and the receptors for IL‐25 (*IL17RB*) and IL‐33 (*IL1RL1*). The isolated ILC2s expressed the expected ILC2‐associated genes *GATA3*, *IL5*, and *IL13*, as well as the receptors *IL17RB* and *IL1RL1*, but showed no detectable expression of *TBX21*, *RORC*, *IFNG*, or *IL17A*, which would indicate the presence of ILC1s and ILC3s, thus supporting ILC2 purity (Figure 1G).

For certain applications, sorting may be desirable to isolate specific ILC2 populations or to enrich for cells with defined characteristics. Thus, to determine whether expanded ILC2s could be flow‐cytometry‐based sorted and recultured, we performed flow‐cytometry‐based sorting at day 21 of expansion. The expanded ILC2s were divided into two fractions: one used for flow‐cytometry‐based cell sorting based on CD45 and CD161 positive expression and the absence of lineage markers, and the other kept as a nonsorted control. After sorting, cells were recultured for an additional 11 days, and their expansion was compared with nonsorted cells from the same donor. No difference in proliferation was observed between sorted and nonsorted ILC2s (Figure 1H), indicating that expanded ILC2s can be flow‐cytometry‐based sorted and subsequently recultured without a major loss in proliferative capacity.

### 3.2. Phenotypic Changes in Human ILC2s During In Vitro Expansion With or Without IL‐25 and IL‐33

Having established a robust expansion protocol, we next examined whether supplementation with IL‐25 or IL‐33 influenced the phenotype of cultured ILC2s. As both cytokines are commonly included in published expansion protocols and are known activators of ILC2s, we compared cell expansion, viability, and expression of activation‐associated markers in cultures maintained with or without IL‐25 or IL‐33. Analyses were performed immediately after isolation (day 0) and after 7, 14, and 21 days of culture.

**Figure 2:**
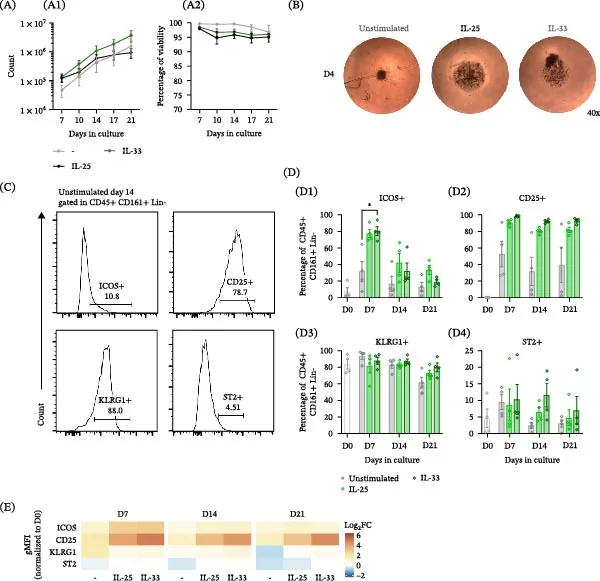
IL‐25 and IL‐33 alter the growth, viability, and activation profile in cultured human ILC2s. (A) ILC2 growth (A1) and viability (A2) from day 7 to 21 in cultures without additional alarmins (‐) or supplemented with either IL‐25 or IL‐33 (*n* = 4 donors for each condition). (B) Representative microscopic images of ILC2s cultured with no alarmins (unstimulated) or with either IL‐25 or IL‐33 on day 4 after isolation (40x magnification). (C) Representative histograms showing the frequency of expression of indicated markers on unstimulated ILC2s. ILC2s were defined as CD45+CD161+Lin‐. Markers analyzed include ICOS, CD25, KLRG1, and ST2. The lineage (Lin) cocktail consisted of CD11c, CD14, CD19, CD303, CD34, CD3, FcεRI, TCRαβ, and TCRγδ. (D) Frequency of ILC2s positive for each analyzed marker (*n* = 4 donors per condition) after culture with no alarmins or in the presence of either IL‐25 or IL‐33. Data are presented as the frequency of CD45+CD161+Lin‐ cells expressing indicated marker. Graphs represent means ± SEM. Significance was tested using a two‐way repeated‐measures ANOVA with Tukey’s post hoc test. (E) Heatmap depicting geometric mean fluorescence intensity (gMFI) of each marker of interest normalized to day 0 in ILC2s cultured either with no alarmins (unstimulated) or with IL‐25 or IL‐33 (*n* = 4 donors per condition). Normalization was performed for each donor individually and is presented as log_2_ fold change relative to day 0. Data are presented as mean ± SEM.  ^∗^ *p* < 0.05.

Supplementation with either IL‐25 or IL‐33 enhanced ILC2 expansion during the first week of culture, although this effect was not maintained at later time points (Figure 2A,B). Cell viability showed a consistent trend towards lower values in IL‐25‐ and IL‐33‐supplemented cultures throughout the culture period, but these differences did not reach statistical significance (Figure 2A). In parallel, both IL‐25 and IL‐33 promoted the expression of the activation markers CD25 (IL‐2RA) and ICOS, with the strongest effects observed after 7 days of culture (Figure 2C–E). KLRG1 expression remained high regardless of culture conditions, whereas ST2 expression increased modestly during the early phase of culture before declining over time, particularly in cultures without IL‐25 or IL‐33 (Figure 2D,E).

Together, these findings suggest that although IL‐25 and IL‐33 transiently enhance early ILC2 expansion, they provide limited benefit for long‐term culture. As efficient expansion was achieved in their absence and their supplementation was associated with increased activation marker expression and a tendency towards reduced viability, omission of IL‐25 and IL‐33 may be advantageous when maintaining a less activated ILC2 phenotype is desired.

### 3.3. Optimization of a Method for Small‐Scale ILC2 Isolation and Expansion

Due to the low frequency of ILC2s in human peripheral blood, isolation and in vitro expansion of these cells typically require large blood volumes. This poses a limitation when working with patient samples, where usually only small amounts of blood are available. Therefore, we sought to establish an ILC2 isolation and expansion method that could be applied to PBMCs obtained from a single 9‐mL tube of blood. Whole blood from female and male healthy donors was collected into heparinized tubes, and PBMCs were isolated as described above. From each sample, 1 × 10^7^ PBMCs were used for ILC2 isolation.

**Figure 3:**
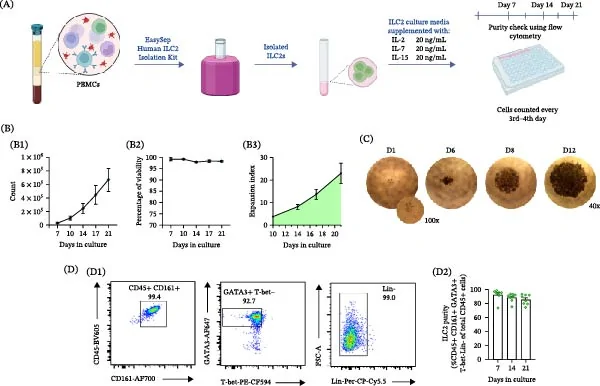
Optimization of a method for small‐scale ILC2 isolation and expansion. (A) Schematic overview of the workflow for ILC2 isolation, in vitro expansion, and analysis from small‐scale blood samples (created with http://BioRender.com). (B) ILC2 growth, viability, and expansion index from day 7 to 21 (day 10–21 for expansion index) for each time point counted (*n* = 8 donors). (C) Representative microscopic pictures of ILC2s at day 1, 6, 8, and 12 after isolation (40x and 100x magnification). (D) Representative gating strategy used for assessment of ILC2 purity at different time‐points during culture. Representative gating from day 14 (D1). Lineage (Lin) cocktail consists of CD11c, CD14, CD19, CD303, CD34, CD3, FcεRI, TCRαβ, and TCRγδ. Quantification of ILC2 frequency in culture at day 7, 14, and 21, defined as frequency of CD45+CD161+GATA3+T‐bet‐Lin‐ cells (*n* = 8 donors) (D2). Data are presented as mean ± SEM.

To maintain the simplicity of the large‐scale method, we used the same isolation kit but reduced the reagent volumes to one‐quarter (1/4) of those used for larger isolations to account for the lower PBMC input (see Table S2 for adjusted volumes). As expected, the yield of ILC2s was lower than that obtained from 2 × 10^8^ PBMCs and ranged between 200 and 600 cells per donor. Using the same expansion conditions as described above, ILC2s expanded ~20‐fold between days 7 and 21, reaching yields of 1 × 10^5^ to 1 × 10^6^ cells. Mean viability remained high at ~98% throughout the culture period (Figure 3A–C), and the mean purity of expanded ILC2s, like those obtained from large‐scale isolations, remained around 90% at all time points (Figure 3D).

In summary, these results demonstrate that our optimized method enables reliable isolation and robust expansion of ILC2s from small blood volumes, making it suitable for studies using limited or rare patient samples.

### 3.4. Isolated ILC2s Retain Stability and Functionality After In Vitro Expansion

After establishing a sorter‐ and feeder‐free method for the isolation and expansion of ILC2s, we next aimed to determine whether the expanded ILC2s could be used for downstream functional assays. To assess functionality, we analyzed proliferation, cytokine production, and expression of surface activation markers in response to the known ILC2 activators IL‐25 and IL‐33. To evaluate the stability of expanded ILC2s, we additionally included cytokines known to promote ILC1 polarization (IL‐12) and ILC3 polarization (IL‐23) [17, 20].

**Figure 4:**
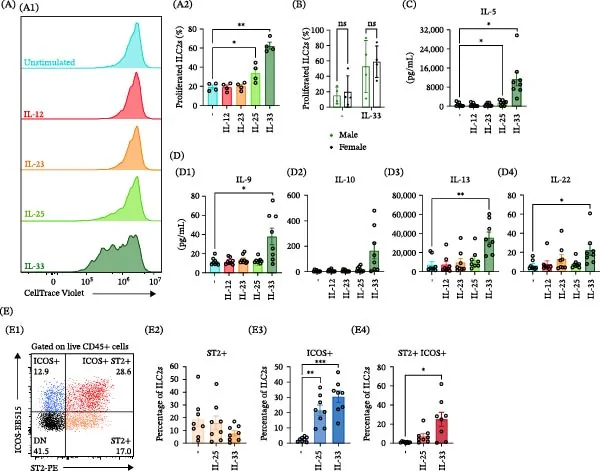
Expanded human ILC2s retain functional responsiveness and phenotypic stability. (A) Proliferation of CellTrace Violet‐labeled ILC2s following stimulation with the ILC‐associated cytokines IL‐12, IL‐23, IL‐25, or IL‐33 for 4 days. Representative CellTrace Violet profiles histograms (A1) and the quantification of the percentage of proliferating cells (A2) are shown for each stimulation (*n* = 4 donors). (B) Percentage proliferation in unstimulated and IL‐33 stimulated cultures comparing male (*n* = 4) and female (*n* = 4) donors. (C) Quantification of IL‐5 production by ELISA and (D) cytokines IL‐9, IL‐10, IL‐13, and IL‐22 by LEGENDplex from supernatants of ILC2s stimulated with either IL‐12, IL‐23, IL‐25, or IL‐33 for 3 days (*n* = 8 donors). (E) Expression of activation marker ICOS on ILC2s left unstimulated or stimulated with either IL‐25 or IL‐33 for 3 days (*n* = 8 donors). Marker expression was assessed as the frequency of ICOS+, ST2+ and ICOS + ST2+ of CD45+ ILC2s. Data are presented as mean ± SEM. Statistical significance for all subfigures except (B) was determined using repeated‐measures one‐way ANOVA with Dunnett’s post hoc test. Statistical differences in (B) were determined using a statistical significance was determined using repeated‐measures two‐way ANOVA with Dunnett’s post hoc test.  ^∗^ *p*  < 0.05,  ^∗∗^ *p*  < 0.01,  ^∗∗∗^ *p*  < 0.001, ns = nonsignificant.

First, we assessed the proliferative response of ILC2s following stimulation with IL‐12, IL‐23, IL‐25, or IL‐33 for 4 days. Consistent with previous studies [16–18], stimulation with IL‐25 and IL‐33 induced a strong proliferative response in vitro (Figure 4A), with comparable IL‐33–induced proliferation observed in ILC2s from male and female donors (Figure 4B). In contrast, ILC2s did not respond to IL‐12 or IL‐23 stimulation.

Next, we determined the cytokine profile in the supernatants from activated ILC2s following stimulation with the indicated cytokines for 3 days. As expected, IL‐33 induced robust production of the classical ILC2‐associated cytokines IL‐5, IL‐9, and IL‐13, compared with other stimuli. Notably, IL‐33 stimulation also induced IL‐22 production by ILC2s (Figure 4C,D). Although IL‐22 production has primarily been attributed to ILC3s, a minor population of IL‐22–producing ILC2s has previously been described by Mjösberg et al. [12]. Stimulation with IL‐25 led to a significant increase in IL‐5 production, while no significant induction of other analyzed cytokines was observed. Neither IL‐12 nor IL‐23 stimulation resulted in a significant cytokine response from cultured ILC2s, although a trend toward increased IFN‐γ production was detected following IL‐12 stimulation (Table S4).

Last, we examined whether ILC2s upregulate the activation marker ICOS upon stimulation with IL‐25 or IL‐33. The expression of the IL‐33 receptor ST2 was included as a control. As shown in Figure 4E, both IL‐25 and IL‐33 stimulation induced ICOS expression in cultured ILC2s. Moreover, IL‐33 stimulation increased the proportion of cells coexpressing ST2 and ICOS, indicative of enhanced IL‐33 responsiveness. Together, these results demonstrate that isolated and expanded ILC2s retain their functional capacity, including proliferative potential and cytokine production, following activation with ILC2‐associated alarmins.

### 3.5. Human ILC2s Are Viable and Functional After Cryopreservation

**Figure 5:**
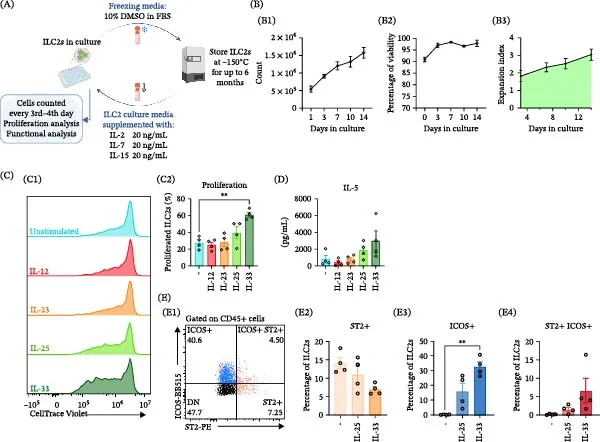
Human ILC2s remain viable and functional following cryopreservation. (A) Schematic overview of the workflow for ILC2 cryopreservation, recovery, and downstream analyses (created with http://BioRender.com). (B) Growth, viability, and expansion index of thawed ILC2s from day 1 to 14 for each time point counted (*n* = 4 donors). Expansion index was calculated from day 3 to 14. (C) Proliferation of CellTrace Violet‐labeled thawed ILC2s following stimulation with IL‐12, IL‐23, IL‐25, or IL‐33 for 4 days. Representative CellTrace Violet histogram profiles (C1) and percentage of proliferating ILC2s (C2) for each stimulation condition quantified. (D) Quantification of IL‐5 production by ELISA in supernatants from thawed ILC2s stimulated with IL‐12, IL‐23, IL‐25, or IL‐33 for 3 days (*n* = 4 donors). (E) Analysis of expression of activation marker ICOS on ILC2s either left unstimulated or stimulated with IL‐25 or IL‐33 for 3 days (*n* = 4 donors). Marker expression was assessed as the frequency of ST2+, ICOS +, and ST2+ICOS + cells among CD45+ ILC2s. Data are presented as mean ± SEM. Statistical significance was determined by using repeated‐measures one‐way ANOVA with subsequent Dunnett’s post hoc test.  ^∗∗^ *p* < 0.01.

Prolonged culture of primary immune cells can lead to cellular senescence, resulting in reduced proliferative capacity, loss of viability, and phenotypic alterations [21, 22]. In addition, patient samples are often obtained on an irregular basis, and experimental resources may not always be immediately available. Cryopreservation therefore provides an effective way to store primary cells for later functional testing. To evaluate whether in vitro‐expanded ILC2s remain viable and functional after cryopreservation, we froze 1 × 10^6^ expanded ILC2s (from day 21 of culture) in 10% DMSO in heat‐inactivated FBS and stored them for up to 6 months. After thawing, ILC2s were cultured using the same method as for freshly isolated cells for up to 14 days, with the cell number and viability assessed every 3–4 days (Figure 5A). Between day 1 and day 14 after thawing, ILC2s expanded approximately threefold, reaching a yield of ~1.5 × 10^6^ cells. As expected, viability was slightly reduced immediately after thawing (91%) but rapidly increased to 98% by day 4 and remained above 97% throughout the culture period (Figure 5B).

To further assess functionality after cryopreservation, we evaluated the proliferative capacity and cytokine production of thawed ILC2s. Using the proliferation assay described above, cells were stimulated with IL‐12, IL‐23, IL‐25, or IL‐33 for 4 days. Similar to freshly expanded ILC2s, thawed ILC2s responded most strongly to IL‐33, which induced significantly increased proliferation (Figure 5C). IL‐25 elicited a modest proliferative response, whereas IL‐12 and IL‐23 had no effect. In parallel, IL‐5 levels were quantified in culture supernatants after 3 days of stimulation with the same activators. Consistent with our observations in freshly isolated ILC2s, IL‐33 induced significantly higher IL‐5 production than the other stimuli (Figure 5D). The expression of activation marker ICOS in thawed ILC2s upon activation with either IL‐25 or IL‐33 was also similar to their fresh counterparts (Figure 5E). Together, these results indicate that in vitro‐expanded and cryopreserved ILC2s retain characteristic IL‐33–driven responsiveness in both proliferation, activation, and type 2 cytokine output.

In summary, these findings demonstrate that in vitro‐expanded human ILC2s can be successfully cryopreserved without major effects on viability or functional responsiveness.

## 4. Discussion

Since their characterization over a decade ago, ILC2s have been recognized as key regulators of type 2 immune responses. They play important roles in tissue homeostasis and repair and orchestrate early immune responses to infection and tissue injury [2, 3, 11]. In addition, ILC2s are implicated in the pathogenesis of several type 2‐associated diseases, including asthma and allergic inflammation [3, 5–7]. However, functional studies of human ILC2s have been challenging due to their low abundance in peripheral blood and the technical limitations of existing isolation and culture methods. In this study, we establish a user‐friendly, scalable, and efficient method for the isolation and in vitro expansion of human ILC2s from both large and small volumes of human peripheral blood. Our method overcomes many limitations of standard approaches, including reliance on feeder cell lines, flow‐cytometry‐based sorting, and the addition of activating cytokines such as IL‐25 and IL‐33 during expansion. This streamlined method reduces complexity and cost while yielding highly viable and functionally responsive ILC2s suitable for downstream experimental applications.

Current isolation protocols for human ILC2s mainly rely on flow‐cytometry‐based cell sorting [13, 15, 17]. Although this approach provides highly pure ILC2s and enables the isolation of specific ILC2 subsets, it also has several drawbacks. First, flow sorting typically requires multiple processing steps, including antibody staining, cell washing, and filtration. The sorting process itself can also be stressful for immune cells and commonly contributes to significant cell loss [23]. As a result, flow‐cytometry‐based isolation of rare cell populations, such as ILC2, often requires large blood volumes, uses large quantities of costly antibodies, and demands extensive hands‐on time. Although pre‐enrichment strategies can shorten sample preparation and sorting time, they do not eliminate the loss of cells inherent to the sorting process. Moreover, these protocols require access to a high‐speed cell sorter and an experienced operator, often for several hours or even a full working day. By contrast, our optimized method provides an easy‐to‐use methodology that allows users to proceed from fresh PBMCs to plated ILC2s in under 3 h. This method also requires minimal technical expertise and no specialized equipment. Furthermore, by avoiding cell sorting altogether, we eliminate sorter‐induced stress and markedly reduce cell loss. These features make the method particularly advantageous for isolating ILC2s from small blood volumes, offering clear benefits when working with clinical patient samples.

Because only small numbers of ILC2s can typically be isolated from human peripheral blood, these cells are commonly expanded in vitro. Although in vitro expansion of primary cells can alter the cellular phenotype and function, it remains the most accepted and widely used strategy to obtain sufficient numbers of human ILC2s for downstream applications [13, 15, 17, 21, 22]. To achieve robust expansion, many protocols rely on feeder cell lines, such as OP9 mouse stromal cells, in combination with complex cytokine cocktails that often include the ILC2‐activating alarmins IL‐25 and IL‐33. This approach can yield up to a 1000‐fold expansion within just a couple of weeks [13, 17]. However, the use of feeder lines introduces additional complexity and potential experimental variability as these cells require specific culture conditions and may be costly to maintain. Feeder‐based systems may also require additional sorting steps to prevent feeder cell contamination in functional assays. We optimized an alternative, feeder‐free method that supports robust expansion, up to 600‐fold, of viable, functionally stable ILC2s over a 21‐day culture period. By using only IL‐2, IL‐7, and IL‐15 during expansion, we avoid prolonged exposure to the activating alarmins IL‐25 and IL‐33, thereby limiting alarmin‐driven activation and associated phenotypic changes in cultured ILC2s [12, 18]. While the absence of IL‐25 and IL‐33 may reduce activation‐associated changes, the expansion protocol still depends on cytokines that support ILC2 survival and proliferation. In particular, omission of IL‐15 markedly impaired expansion in our system, highlighting its importance for efficient long‐term culture. Consistent with this observation, IL‐15 has been incorporated together with IL‐2 and IL‐7 in previously published protocols for the expansion of human ILC2s [24].

A further advantage of our method is its ability to isolate and expand ILC2s from small blood volumes, making it particularly suitable for patient‐based studies. While most ILC2 isolation protocols depend on large‐volume leukapheresis products or buffy coats, resources often unavailable in clinical settings, we demonstrate that ILC2s can be reliably recovered and expanded from a single clinical tube of blood. Despite the lower starting cell numbers (200–600 cells), cultures expanded to ~1 × 10^6^ cells over 21 days, achieving purity and viability comparable to those obtained from larger blood inputs. This capability greatly enhances the feasibility of studying ILC2s from rare patient cohorts, such as individuals carrying specific genetic variants or those with chronic disease, as well as from vulnerable populations, including pediatric or geriatric patients, where blood draw volume is limited.

Successful cryopreservation of primary cells provides researchers with the flexibility to plan and coordinate experiments as needed. Here, we show that expanded ILC2s can be successfully cryopreserved and subsequently thawed and recultured. While primary cells often lose viability or functionality following freeze–thaw cycles, our cryopreserved hILC2s retained high viability (91%–98%), expanded upon reculture, and responded robustly to IL‐33 and IL‐25 stimulation. These findings demonstrate that expanded ILC2s can be cryopreserved and reused for future experiments. Such flexibility is invaluable for longitudinal studies, batch standardization, and collaborative research requiring synchronized experimental workflows. This is of particular interest given recent evidence suggesting that in vitro‐expanded human ILC2s can mediate antitumor activity after adoptive transfer, supporting their proposed use in cancer immunotherapy [24, 25].

Compared with the current published protocols (see Table S6 for method comparison), our method provides several practical and conceptual advantages for the study of human ILC2s. By eliminating the need for flow‐cytometry‐based cell sorting, it avoids the time, cost, and technical expertise associated with multicolor sorting, and its feeder‐free culture system reduces potential artifacts arising from coculture‐dependent activation. Indeed, the EasySep Human ILC2 Isolation Kit enabled the generation of highly pure ILC2s within 2.5 h, representing a substantial simplification compared with previously reported approaches that require expansion of mixed ILC populations, followed by additional magnetic enrichment or flow cytometric sorting to obtain pure ILC2s. Furthermore, omission of IL‐25 and IL‐33 supplementation minimized the induction of activation‐associated markers while still supporting robust cell expansion, resulting in ILC2 cultures with a lower activation state. Additionally, compatibility with small‐volume samples aligns well with the constraints of clinical studies, and the method supports efficient cryopreservation, enabling long‐term storage and flexible downstream use. Together, these features make the method accessible, reproducible, and broadly applicable for both basic and translational investigations into human ILC2 biology. It should also be acknowledged that our method, due to the use of CRTH2‐based positive selection, restricts isolation to CRTH2‐positive ILC2s. Although CRTH2 remains the most reliable and widely accepted marker for identifying circulating human ILC2s, this strategy inherently excludes potential ILC2 subsets that lack or downregulate CRTH2 under specific inflammatory or tissue‐derived conditions [4, 12, 26–28]. Importantly, this constraint reflects the isolation method rather than the expansion system itself as the culture method can be applied equally well to flow‐sorted populations when broader inclusion of ILC2 phenotypes is desired. Furthermore, a limitation of our method is the relatively low initial yield of ILC2s, particularly from small amounts of whole blood, which, of course, can induce sample variability and longer expansion times.

In summary, this study provides a robust and simplified method for the isolation, expansion, and preservation of functional human ILC2s from both large‐ and small‐volume whole blood. Our approach offers an accessible alternative to existing protocols by eliminating the need for flow‐cytometer‐based sorting, feeder cells, and activating cytokines, while maintaining cell purity, viability, and functionality. Moreover, the ability to cryopreserve and subsequently reactivate ILC2s introduces valuable flexibility for various experimental and clinical applications. Collectively, this platform provides opportunities for mechanistic ILC2 research, disease modeling, and the development of patient‐specific immunological studies.

## Author Contributions

Mattias N. D. Svensson conceived, designed, and supervised the research and wrote the manuscript. Lina K. Andersson performed experiments, analyzed data, and wrote the manuscript. Miriam Bollmann contributed to the acquisition and analysis of data and wrote the manuscript. Agnieszka Lastowska, Isabel Haupt, Lisi Wagener, and Anders Nguyen contributed to the acquisition of data. Johan Svensson and Linda C. Johansson provided tools and reagents and key scientific input.

## Funding

This work was supported by the Swedish Research Council (Grants 2025‐03125 and 2021‐00997 to Mattias N. D. Svensson and Grants 2019‐01891 and 2025‐02420 to Linda C. Johansson), the Swedish Society for Medical Research (Grant S19‐0062 to Mattias N. D. Svensson, Grant S19‐0121 to Linda C. Johansson, and Grant PG‐23‐0379‐H‐01 to Miriam Bollmann), the Foundation for Research in Rheumatology (FOREUM) to Mattias N. D. Svensson, The Swedish Rheumatology Association to Mattias N. D. Svensson and Miriam Bollmann, The Sahlgrenska Academy to Mattias N. D. Svensson, the ALF‐agreement (Grant ALFGBG‐1028985 to Mattias N. D. Svensson), the King Gustaf V’s 80th Anniversary Foundation to Mattias N. D. Svensson and Miriam Bollmann, the Professor Nanna Svartz Research Foundation to Mattias N. D. Svensson and Miriam Bollmann, the Ollie and Elof Ericssons Foundation to Mattias N. D. Svensson, the Apotekare Hedberg Foundation to Mattias N. D. Svensson, The Crafoord Foundation to Miriam Bollmann and Johan Svensson, the Åke Wiberg Foundation to Mattias N. D. Svensson, the Gunvor och Josef Anérs Foundation to Mattias N. D. Svensson, the IngaBritt och Arne Lundbergs Research Foundation to Mattias N. D. Svensson, the Ragnar Söderberg Foundation (Grant 7/22‐A to Linda C. Johansson), the Swedish Foundation for Strategic Research (Grant FFL18‐0182 to Linda C. Johansson), and the Knut and Alice Wallenberg Foundation (Grant 2021.0065 to Linda C. Johansson).

## Disclosure

All authors approved the submitted version of the manuscript.

## Conflicts of Interest

The authors declare no conflicts of interest.

## Supporting Information

Additional supporting information can be found online in the Supporting Information section.

## Supporting information


**Supporting Information** Table S1: List of materials used in experimental procedures. Table S2: Volumes of EasySep Human ILC2 Isolation Kit (STEMCELL Technologies) components used for large‐scale and small‐scale isolation of human ILC2s from PBMCs obtained using SepMate PBMC Isolation Tubes (STEMCELL Technologies). Table S3: Sequences of primers used for qPCR. Table S4: Concentrations of cytokines (pg/mL) IFN‐G, IL‐17A, IL‐17F, and IL‐4 in supernatants from ILC2s stimulated with either IL‐12, IL‐23, IL‐25, or IL‐33 for 3 days (*n* = 8). Table S5: Geometric mean fluorescence intensity (gMFI) of markers of interest in ILC2s cultured in the absence (unstim) or presence of IL‐25 or IL‐33 and analyzed at days 0, 7, 14, and 21. Table S6: Comparing methods of isolating and expanding human ILC2s. Figure S1: Fluorescence minus one (FMO) controls used to define positive and negative staining populations for each marker included in the ILC2 purity subfigure. Figure S2: Representative flow cytometry gating strategy used for assessment of ILC2 purity. Figure S3: Fluorescence minus one (FMO) controls used to define positive and negative staining populations for each marker included in the activation phenotyping subfigure. Figure S4: Representative flow cytometry gating strategy used for assessment of activation‐associated marker expression in ILC2s before and after culture under different conditions. Figure S5: Representative flow cytometry gating strategy used for analysis of ILC2 proliferation.

## Data Availability

All data needed to evaluate the conclusions in the paper are present in the paper and/or the Supporting Information.
