# Supplementary material for: An Optimized Feeder‐Free Method for Isolation and Expansion of Functional ILC2s Isolated From Human Peripheral Blood
Source: J Immunol Res. 2026 Aug 27;2026:7688318. doi: 10.1155/jimr/7688318 (PMC13522718; doi:10.1155/jimr/7688318)
Supplement: Supplementary file 1 [file JIMR-2026-7688318-s001.docx]

**Supplementary Material**

**An optimized feeder-free method for isolation and expansion of functional ILC2s isolated from human peripheral blood**

Lina K. Andersson^1*^MSc, Miriam Bollmann^1*^ PhD, Agnieszka Lastowska^1^ MSc, Isabel Haupt^1^ BSc, Lisi Wagener^1^ MSc, Anders Nguyen^1^ MSc, Johan Svensson^2^ MD PhD, Linda C. Johansson^3,4^ PhD, Mattias N.D. Svensson^1^ PhD

^1^Department of Rheumatology and Inflammation Research, Sahlgrenska Academy, Institute of Medicine, University of Gothenburg, Gothenburg 41346, Sweden

^2^Department of Paediatrics, Skåne University Hospital, Lund University, Malmö, Sweden

^3^Department of Medical Biochemistry and Cell biology, Sahlgrenska Academy, Institute of Biomedicine, University of Gothenburg 41390, Sweden

^4^SciLifeLab, Institute of Biomedicine, University of Gothenburg, Gothenburg, Sweden

*These authors contribute equally

Correspondence to Mattias N.D. Svensson, Department of Rheumatology and Inflammation Research, Sahlgrenska Academy, Institute of Medicine, University of Gothenburg, Gothenburg 41346, Sweden, Mattias.svensson@rheuma.gu.se

**Supplementary Table S1.** List of materials used in experimental procedures.

| Reagent or Resource | Source | Identifier |
| --- | --- | --- |
| Antibodies |  |  |
| Anti-human CD11c-PerCp5.5 (dilution 1:40) | BD | 565227 (clone: B-Ly6) |
| Anti-human CD14-PerCp5.5 (dilution 1:40) | BD | 561116 (clone: M5E2) |
| Anti-human CD161-AF700 (dilution 1:40) | BioLegend | 339941 (clone: HP-3G10) |
| Anti-human CD19-PerCp5.5 (dilution 1:40) | BioLegend | 302230 (clone: HIB19) |
| Anti-human CD25-APC-H7 (dilution 1:40) | BD | 560244 (clone: M-A251) |
| Anti-human CD303-PerCp5.5 (dilution 1:40) | BioLegend | 354209 (clone: 2O1A) |
| Anti-human CD34-PerCp5.5 (dilution 1:10) | BioLegend | 343611 (clone: .561) |
| Anti-human CD3-PerCp5.5 (dilution 1:40) | BD | 560835 (clone: UCHT1) |
| Anti-human CD45-BV605 (dilution 1:40) | BioLegend | 304133 (clone: HI30) |
| Anti-human FcεRI-PerCp5.5 (dilution 1:40) | BioLegend | 334621 (clone: AER-37 (CRA-1)) |
| Anti-human GATA3-AF647 (dilution 1:40) | BD | 560078 (clone: L50-823 ) |
| Anti-human ICOS-BB515 (dilution 1:40) | BD | 564549 (clone: DX29) |
| Anti-human KLRG1-BV421 (dilution 1:40) | BD | 568269 (clone: Z7-205.rMAb) |
| Anti-human ST2-PE (dilution 1:200) | BD | 569315 (clone: U33-374) |
| Anti-human Tbet-PE-CF594 (dilution 1:40) | BioLegend | 562467 (clone: O4-46) |
| Anti-human TCRαβ-PerCp5.5 (dilution 1:40) | BioLegend | 306723 (clone: IP26) |
| Anti-human TCRγδ-PerCp5.5 (dilution 1:200) | BD | 564157 (clone: B1) |
| Fixable Viability Dye eFluor™ 506 (dilution 1:1000) | Invitrogen | 50246097 |
| Annexin V-FITC (dilution 1:40) | BD | 556547 |
| 7-AAD (dilution 1:40) | BD | 559925 |
| CellTrace™ Violet | Invitrogen | C34571 |
| eBioscience™ Foxp3 / Transcription Factor Staining Buffer Set | Invitrogen | 00-5523-00 |
| Culture reagents and cytokines |  |  |
| Recombinant human IL-2 | BioLegend | 589104 |
| Recombinant human IL-7 | BioLegend | 581904 |
| Recombinant human IL-12 | Peprotech | 200-12H |
| Recombinant human IL-15 | R&D Systems | 247-ILB-025 |
| Recombinant human IL-23 | Peprotech | 200-23 |
| Recombinant human IL-25 | Peprotech | 200-24 |
| Recombinant human IL-33 | BioLegend | 581804 |
| Human AB serum | Sigma-Aldrich | H3667 |
| MEM α GlutaMax™ | Gibco | 32561037 |
| Penicillin/Streptomycin | Gibco | 15070063 |
| 2-mercaptoethanol (50 mM) | Gibco | 21985-023 |
| Fetal Bovine Serum | Cytiva | SV30160.03 |
| Dimethyl Sulfoxide | Corning | 25-950-CQC |
| Gentamicin | Gibco | 15750045 |
| DPBS, no calcium, no magnesium | Gibco | 14190136 |
| EDTA (0.5 M), pH 8.0, RNase-free | Invitrogen | AM9260G |
| Commercial assays |  |  |
| EasySep™ Human ILC2 Isolation Kit | STEMCELL | 17782 |
| ELISA MAX™ Standard Set Human IL-5 | BioLegend | 430401 |
| iScript cDNA synthesis kit | Bio-Rad | 1708890 |
| LEGENDplex™ HU Th Cytokine Panel (12-plex) | BioLegend | 741028 |
| Total RNA Purification Plus Micro Kit | Norgen | 48500 |
| Other |  |  |
| Corning® CoolCell™ FTS30 | Corning | CLS432009 |
| Corning® Falcon® Round Bottom Test Tubes | Corning | CLS352235 |
| CryoPure Tube 1.8 mL Blue | Sarstedt | 72.379.005 |
| EasySep™ Magnet | STEMCELL | 18000 |
| Ficoll-Paque™ density gradient media | Cytiva | 17144003 |
| MicroAmp™ Fast Optical 384-Well Reaction Plate | Applied Biosystems | 4343370 |
| SepMate™ PBMC Isolation Tubes | STEMCELL | 85450 |
| SsoAdvanced Universal SYBR Green Supermix | Bio-Rad | 1725274 |
| Sterile Nunclon Delta Surface round-bottom 96-well plate | Thermo Scientific | 163320 |
| Sulfuric Acid | In house |  |
| TMB | Thermo Scientific | 34028 |
| UltraPure™RNAase and DNAse-Free Distilled Water | Invitrogen | 10977035 |

**Supplementary Table S2.** Volumes of EasySep™ Human ILC2 Isolation Kit (STEMCELL Technologies) components used for large-scale and small-scale isolation of human ILC2s from PBMCs obtained using SepMate™ PBMC Isolation Tubes (STEMCELL Technologies). For all steps not listed in this table, the original volumes specified in the manufacturer’s protocol were used.

| Component Name | Component # | Large scale (1mL of sample) | Small-scale (250µL of sample)* |
| --- | --- | --- | --- |
| Anti-Human CD32 (Fc gamma RII) Blocker for Positive Selection | 18520 | 100 µL | 25 µL |
| Human CRTH2-PE Positive Selection Cocktail | 17782CA | 10 µL | 2.5 µL |
| Release PE Positive Selection Cocktail | 17654C | 100 µL | 25 µL |
| Releasable RapidSpheres™ | 50201 | 100 µL | 25 µL |
| Release Buffer | 20145 | 100 µL | 25 µL |
| Human ILC2 Isolation Cocktail | 17782CB | 50 µL | 12.5 µL |
| Dextran RapidSpheres™ | 50103 | 50 µL | 12.5 µL |

**Supplementary Table S3.** Sequences of primers used for qPCR. *Housekeeping gene.

| Primer | Forward 5’-3’ | Reverse 5’-3’ |
| --- | --- | --- |
| *GAPDH** | TCGGAGTCAACGGATTTG | CAACAATATCCACTTTACCAGAG |
| *GATA3* | AAAATGAACGGACAGAACC | GGGGTCTGTTAATATTGTGAAG |
| *IFNG* | GGTAACTGACTTGAATGTCC | TTTTCGCTTCCCTGTTTTAG |
| *IL13* | ATCACCCAGAACCAGAAG | ATGCAAGCTGGAAAACTG |
| *IL17a* | ATGCAAGCTGGAAAACTG | CTGTGAGCTCTTTCCTTATG |
| *IL5* | AACTGTGCACTGAAGAAAT | CTAGGAATTGGTTTACTCTCC |
| *RORC* | TTTTGAAGGCAAATACGGTG | AGTGGGAGAAGTCAAAGATG |
| *TBX21* | CTTTCCAAGAAACCCAGTTC | GTCAACAGATGTGTACATGG |
| *IL17RB* | GCTGACTCCATATTTTCCTAC | GTTGTTATCCAGAGGGAAAG |
| *IL1RL1* | CACCTCTTGAGTGGTTTAAG | TTGGCTCCATTTTCATTGTG |

**Supplementary Table S4.** Concentrations of cytokines (pg/mL) IFNγ, IL-17A, IL-17F, and IL-4 in supernatants from ILC2s stimulated with either IL-12, IL-23, IL-25 or IL-33 for 3 days (n=8). Cytokine levels determined using the LEGENDplex™ HU Th Cytokine Panel (12-plex) Kit. Data are presented as Mean ± SEM.

| Cytokine | Unstimulated | IL-12 | IL-23 | IL-25 | IL-33 |
| --- | --- | --- | --- | --- | --- |
| IFNγ | 7.80 ± 1.40 | 172.60 ± 80.96 | 10.87 ± 2.28 | 8.45 ± 1.29 | 85.69 ± 34.07 |
| IL-17A | 1.82 ± 0.47 | 1.79 ± 0.23 | 2.25 ± 0.52 | 1.72 ± 0.15 | 2.07 ± 0.22 |
| IL-17F | 5.44 ± 0.17 | 5.38 ± 0.06 | 5.56 ± 0.17 | 5.40 ± 0.05 | 5.58 ± 0.08 |
| IL-4 | 119.80 ± 63.87 | 98.08 ± 45.55 | 148.10 ± 75.58 | 100.4 ± 35.73 | 187.4 ± 55.21 |

**Supplementary Table S5.** Geometric mean fluorescence intensity (gMFI) of markers of interest in ILC2s cultured in the absence (Unstim) or presence of IL-25 or IL-33 and analysed at days 0, 7, 14, and 21. ILC2s were defined as CD45+CD161+Lin- cells. Markers analysed included ICOS, CD25, KLRG1 and ST2. The lineage (Lin) cocktail consisted of CD11c, CD14, CD19, CD303, CD34, CD3, FcεRI, TCRαβ, and TCRγδ. Data are presented as Mean ± SEM.

| Marker | D0 | D7 Unstim | D7 IL25 | D7 IL33 | D14 Unstim | D14 IL25 | D14 IL33 | D21 Unstim | D21 IL25 | D21 IL33 |
| --- | --- | --- | --- | --- | --- | --- | --- | --- | --- | --- |
| ICOS | 2318 ± 482 | 6262 ± 1146 | 5108 ± 834 | 3429 ± 586 | 5318 ± 551 | 3901 ± 192 | 3309 ± 431 | 12750 ± 1442 | 14027 ± 1774 | 4893 ± 914 |
| CD25 | 4882 ± 358 | 46463 ± 3467 | 94733 ± 17151 | 17534 ± 7446 | 50830 ± 12275 | 104019 ± 16432 | 24196 ± 12004 | 72879 ± 9003 | 139757 ± 6685 | 26953 ± 11583 |
| KLRG1 | 8808 ± 1459 | 12260 ± 1037 | 13973 ± 1908 | 12113 ± 1651 | 9255 ± 1261 | 11332 ± 2110 | 6358 ± 1054 | 13283 ± 3079 | 15453 ± 2887 | 29918 ± 8056 |
| ST2 | 2836 ± 446 | 2557 ± 182 | 3081 ± 320 | 2020 ± 282 | 2246 ± 142 | 2466 ± 359 | 1866 ± 285 | 2528 ± 368 | 2785 ± 358 | 2662 ± 222 |

**Supplementary Figure S1. Fluorescence Minus One (FMO) controls used to define positive and negative staining populations for each marker included in the ILC2 purity panel**. Markers analysed included Live/Dead, CD45, CD161, GATA3, T-bet, and the lineage (Lin) cocktail, consisting of CD11c, CD14, CD19, CD303, CD34, CD3, FcεRI, TCRαβ, and TCRγδ. The orange dotted line indicates the threshold used to distinguish negative and positive populations

**Supplementary Figure S2. Representative flow cytometry gating strategy used for assessment of ILC2 purity.** Gating was performed on ILC2s isolated from buffy coat and analysed after 14 days of culture. Markers analysed included Live/Dead, CD45, CD161, GATA3, T-bet, and the lineage (Lin) cocktail, consisting of CD11c, CD14, CD19, CD303, CD34, CD3, FcεRI, TCRαβ, and TCRγδ. ILC2s were defined as CD45+CD161+GATA3+T-bet-Lin- cells.

**Supplementary Figure S3. Fluorescence Minus One (FMO) controls used to define positive and negative staining populations for each marker included in the activation phenotyping panel.** Markers analysed included Live/Dead, CD45, CD161, KLRG1, CD25, ICOS, ST2, and the lineage (Lin) cocktail, consisting of CD11c, CD14, CD19, CD303, CD34, CD3, FcεRI, TCRαβ, and TCRγδ. The orange dotted line indicates the threshold used to distinguish negative and positive populations.

**Supplementary Figure S4. Representative flow cytometry gating strategy used for assessment of activation-associated marker expression in ILC2s before and after culture under different conditions**. Representative gating was performed on ILC2s isolated from buffy coat and analysed after 14 days of culture in the absence of alarmins IL-25 or IL-33. Markers analysed included Live/Dead, CD45, CD161, ICOS, CD25, KLRG1, ST2, and the lineage (Lin) cocktail, consisting of CD11c, CD14, CD19, CD303, CD34, CD3, FcεRI, TCRαβ, and TCRγδ. ILC2s were defined as CD45+CD161+Lin- cells.

**Supplementary Figure S5. Representative flow cytometry gating strategy used for analysis of ILC2 proliferation.** ILC2s were cultured for 4 days either without stimulation or in the presence of IL-25 or IL-33. The representative gating strategy shown corresponds to IL-33-stimulated ILC2s. Markers analysed included Live/Dead and CellTrace™ Violet. Proliferating cells were identified based on dilution of CellTrace™ Violet fluorescence.

^1^Lim, A. I., Menegatti, S., Bustamante, J., Le Bourhis, L., Allez, M., Rogge, L., Casanova, J. L., Yssel, H., & Di Santo, J. P. (2016). IL-12 drives functional plasticity of human group 2 innate lymphoid cells. The Journal of experimental medicine, 213(4), 569–583. <https://doi.org/10.1084/jem.20151750>

^2^ Li, Z., Ma, R., Tang, H., Guo, J., Shah, Z., Zhang, J., Liu, N., Cao, S., Marcucci, G., Artis, D., Caligiuri, M. A., & Yu, J. (2024). Therapeutic application of human type 2 innate lymphoid cells via induction of granzyme B-mediated tumor cell death. Cell, 187(3), 624–641.e23. <https://doi.org/10.1016/j.cell.2023.12.015>

^3^ Ciancaglini, C., Santopolo, S., Vacca, P., Moretta, L., & Quatrini, L. (2026). Protocol to isolate, expand, and activate human type 2 innate lymphoid cells to study immune checkpoint function. STAR protocols, 7(2), 104461. Advance online publication. <https://doi.org/10.1016/j.xpro.2026.104461>

| **Method** | **Patient Info** | **Time cultured** | **Co-culture** | **Cytokines used for expansion** | **PBMCs used** | **Isolated ILC2s** | **Yield** | **Fold expansion** | **Viability** | **Culture Purity** | **Source** |
| --- | --- | --- | --- | --- | --- | --- | --- | --- | --- | --- | --- |
| Magnet-based selection of ILC2s (large-scale) | Healthy Donors | 21 days | None | IL-2, IL-7, IL-15 | 200 x 10^6 | 2,000-3,000 | 1 x 10^5 to 2 x 10^6 | ~200-1,000 | 97-99% | >90% | Current Paper |
| Magnet-based selection of ILC2s (small-scale) | Healthy Donors | 21 days | None | IL-2, IL-7, IL-15 | ~10^6 | 200-600 | 1 x 10^5 to 1 x 10^6 | ~200-5,000 | 97-99% | >90% | Current Paper |
| Magnet-based depletion of Lin+ cells + Sorting | Crohn's Patients on active medication | 7 or 14 days | Irridated PBMCs or OP9 | IL-2, IL-7 | NA | NA | NA | ~5,000 | NA | NA | ^1^ Lim, A et al. (2016) |
| Magnet-based selection of NK cells then ILCs + Sorting | Healthy Donors | 28 days | OP9 | IL-2, IL-7, IL-15 | 10 mL LRS blood | Unknown due to initial expansion of all ILCs | 20 to 100 x 10^6 | NA | NA | NA | ^2^ Li, Z et al. (2024) |
| Magnet-based depletion of non-ILC2s + Magnet-based selection of ILC2s | Healthy Donors | 14 days | None | IL-2, IL-7, IL-1β | 3.5 to 4.5 x 10^6 | Unknown due to initial expansion of unpure ILC2s (~66% pure) the first 10 days | 1 to 5 x 10^6 | NA | NA | 95% | ^3^ Ciancaglini, C et al. (2026) |

**Supplementary Table S6.** Comparing methods of isolating and expanding human ILC2s. LRS: Leukocyte reduction system, NA: Not indicated.
